# Corneal Diseases and Corneal Transplantation: Current Updates and Perspectives

**DOI:** 10.3390/jcm14248678

**Published:** 2025-12-07

**Authors:** Nir Erdinest, Itay Lavy

**Affiliations:** Department of Ophthalmology, Hadassah-Hebrew University Medical Center, Faculty of Medicine, Hebrew University of Jerusalem, Jerusalem 91120, Israel

Recent years have brought significant changes in how we treat corneal disease, thanks to advances in immunology, regenerative medicine, surgical techniques, and new drug therapies. Corneal transplantation is still the most common type of solid tissue transplant worldwide, and approaches to managing rejection, preventing infection, and tailoring surgery to individual patients are evolving rapidly to meet the growing need for better vision restoration [1,2]. When we use targeted immunosuppressive treatments, especially for patients at higher risk of graft rejection, we see fewer cases of corneal allograft rejection and better long-term outcomes, particularly with newer biologics and cell-based therapies. Among the most important recent developments are Rho kinase (ROCK) inhibitors, which have changed how we approach corneal endothelial dystrophies and persistent corneal edema. These drugs work by helping endothelial cells function better, reducing the need for full-thickness transplants, and making it possible to use less invasive procedures [3].

Recent progress in diagnostic tools and artificial intelligence is helping us classify diseases more accurately and predict outcomes in complex cases, which allows better patient matching with specific treatments. Surgery is moving toward more personalized, less invasive procedures that use detailed imaging and biomechanical analysis to guide decisions, rather than using the same approach for everyone. Better understanding of how the ocular surface stays healthy, how wounds heal, and how the body fights infections has led to improved prevention and treatment strategies for both infectious and inflammatory corneal problems. Together, these advances are improving outcomes for patients dealing with corneal blindness or dysfunction, as shown in recent literature and in the studies presented in this Special Issue [4,5,6].

This Special Issue brings together these recent advances through different studies on pharmacological, surgical, infectious, and immunological aspects of corneal care. The papers included here connect discoveries in the laboratory with what actually happens in the clinic, presenting new ways of thinking about long-standing problems and new opportunities in treating corneal disease.

Starting with pharmacological approaches, the first study examines how the ROCK inhibitor Ripasudil works in real clinical practice for treating corneal edema from various causes. Drawing on data from a large group of patients studied after their treatment, the authors look at how well this drug works in actual practice and whether it is safe. The study shows how moving away from traditional supportive care toward regenerative drug therapy can improve outcomes, especially for patients with persistent swelling that does not respond to standard treatments. The results show that Ripasudil can reduce corneal thickness and improve how well patients see, making it a practical option for a wide range of corneal edema problems. This research demonstrates how a drug discovered in the laboratory can become a useful tool for eye doctors, expanding the options available for patients with corneal endothelial problems (Contribution 1).

Moving to surgical innovations, a second paper evaluates femtosecond laser-assisted arcuate keratotomy for treating astigmatism that stays after keratoplasty. Even when corneal transplants work well, patients often have leftover astigmatism that limits how well they can see. The authors look at how this laser technique helps fix this problem and whether it improves vision without needing glasses. The study explains how the laser makes precise cuts in the cornea to reshape it, correcting the astigmatism and helping transplant patients see better. These findings show how advanced technology can be used in a straightforward way to solve a common problem after transplant surgery, giving patients a chance to gain better vision after their recovery (Contribution 2).

Turning to infections, which present ongoing challenges in corneal care, a third investigation compares keratitis that comes back with first-time infections in a very large group of patients. The authors look at what causes infection to return, which patients are at higher risk, and what happens to their vision over time. Their analysis shows which factors make it more likely that an infection will come back and what doctors can do to prevent this. The findings stress the need for solid prevention strategies, catching infections early, and adjusting how we treat patients to break the cycle of repeated infection and preserve vision. The paper brings together large numbers of patient cases with practical lessons from daily eye care practice, helping doctors understand and manage corneal infections better (Contribution 3).

Looking at immunological aspects, a fourth study focuses on inflammation that is hidden below the surface in corneal scars that look fine to the eye. Even when scars appear clinically inactive, the authors find ongoing immune activity under the microscope that routine clinical assessment would miss. Their work shows that relying only on what we see clinically can cause us to overlook ongoing processes that might lead to graft failure or other problems after transplant. The distinction between appearing inactive clinically and truly having no immune activity is important for planning surgery and ensuring the graft survives long-term. The findings emphasize why detailed tissue analysis matters when assessing candidates for transplant, opening up new possibilities for personalizing immunosuppression and follow-up care for corneal transplant patients (Contribution 4).

Looking at complex surgical challenges, the final contribution describes a rare but important case: managing intraocular lens dislocation in Brown–McLean syndrome. This syndrome causes peripheral corneal swelling that may develop years after cataract surgery or IOL implantation, and lens dislocation in this setting presents a difficult surgical problem. The authors show step-by-step how they reattached the lens while protecting the corneal endothelium and keeping surgical trauma to a minimum. This case shows how eye doctors have become better at planning personalized treatments and using gentle surgical approaches to handle problems that appear in specific patient groups. The technique described here adds to the growing toolkit of ways to manage IOL problems, showing how important it is to treat each patient as an individual and adapt surgical methods to each situation (Contribution 5).

## Future Directions and Clinical Implications

The research brought together in the current Special Issue shows where corneal disease management stands today, confirming that we need teamwork across different specialties and willingness to change as clinical practice develops. Moving forward will require continuing to combine targeted drug therapy, new surgical techniques, and immune system management. Personalizing immunosuppression, making more regenerative drugs available, and using computer programs to help eye-care practitioners make decisions will help us close the gap between what we understand about disease and actual benefits for patients. Just as importantly, making sure advances reach all populations fairly, adapting them to places with fewer resources, and testing long-term safety carefully will be essential to ensure that improvements in care last and reach patients everywhere in different healthcare settings.
